# Flight performance of great cormorants *Phalacrocorax carbo sinensis* suggests sufficient muscle capacity for adaptive speed adjustment

**DOI:** 10.1242/jeb.251056

**Published:** 2026-01-02

**Authors:** Anders Hedenström, Marco KleinHeerenbrink, Susanne Åkesson

**Affiliations:** ^1^ Department of Biology, Lund University, 223 62 Lund, Sweden; ^2^ Department of Biology, University of Oxford, Life and Mind Building, South Parks Road, Oxford OX1 3EL, UK

**Keywords:** Aerodynamics, Constraint, Wind compensation, Flock formation, Flight–leg muscle trade-off, Flight mechanics

## Abstract

Power required to fly for a bird generally follows a U-shaped function of airspeed, with higher cost at both low and high speeds. Because power required increases with body mass faster than power available from flight muscles, larger birds may experience restricted flight speed ranges and climbing capabilities. Previous studies found limited flight performance in cormorants. Adapted for both flight and sub-surface swimming, they trade off larger flight muscles for powerful leg muscles used for diving. Our study tested whether the flight performance of greater cormorants is constrained by measuring airspeed under various seasonal and wind conditions. If flight muscles severely limit the range of flight speeds, cormorants would not be able to adopt ecologically relevant speeds between seasons and not increase speed in headwinds to minimize cost of transport. Results suggest that cormorants can achieve airspeeds beyond minimum power speed, selecting speeds near maximum range during autumn migration and exceeding this range on spring migration and during foraging flights. However, expected speed adjustments to headwinds were inconsistent, with some situations lacking the anticipated responses. The cormorants demonstrated partial wind drift compensation by adjusting flight headings along coastlines, though airspeed adjustments were not always observed. Although greater cormorants appear capable of reaching ecologically relevant speeds in many contexts, the overall scope of their flight speeds remains relatively narrow compared with smaller bird species. These findings indicate that greater cormorants have muscle power for adaptive behaviour in some cases, despite the influence of physiological constraints on their flight performance.

## Teaser

**Summary:** Contrary to previous assumptions, greater cormorants have sufficient muscle power to adjust flight speed in relation to ecological context and winds.

## INTRODUCTION

Animal flight is a complex phenomenon shaped by physical laws, ecological pressures and physiological constraints, with energetics playing a central role in understanding its function and evolution. The power required to fly (*P*) in birds follows a theoretical U-shaped function in relation to airspeed (*V*) (Pennycuick, 1968; Tucker, 1973), a relationship that has received empirical support (e.g. Tucker, 1968; Tobalske et al., 2003; Askew and Ellerby, 2007; Engel et al., 2010; Hedenström, 2025). Given this power–speed relationship, *P*(*V*), and considering realistic currency assumptions, ecological contexts and associated objectives of the flight, several ‘optimal’ flight speeds can be derived (Hedenström and Alerstam, 1995). For example, when the objective is to minimize the energy cost per unit time, the optimal speed is given by d*P*/d*V*=0 and is denoted as the minimum power speed *V*_mp_. As the term implies, any speed above the minimum power speed requires more power. However, if the objective is to minimize the energy cost per unit distance (i.e. cost of transport), the optimal speed is the maximum range speed *V*_mr_ given by d*P*/d*V*=*P*/*V*. In addition to these two fundamental optimal speeds, alternative optimal speeds can be derived that are associated with maximum energy delivery rate to a nest (Norberg, 1981) and optimal speed associated with time-selected migration (Hedenström and Alerstam, 1995), which both depend on the rates of energy acquisition and fuel deposition at feeding areas and migratory stopovers, respectively. The maximum range speed is higher than the minimum power speed by a factor of approximately 1.32 (Pennycuick, 2008), and speeds of maximum energy delivery rate and time-selected migration are higher still and depend on the rates of energy acquisition/fuel accumulation (Hedenström and Alerstam, 1995).

We expect that observable flight speeds are the result of a combination of physical, behavioural and evolutionary/morphological factors. For level steady flight, we can derive a power curve from physical principles that is primarily dependent on flight speed, body mass and morphology of the bird (Pennycuick, 1968). From this power curve we can derive characteristic speeds corresponding to various physical properties and ecological objectives. The flight speed of birds is expected to depend on: air density (altitude), which directly affects the power curve (Hedenström et al., 2002); wind speed, which affects the transport cost (Pennycuick, 1978); vertical speed, which deviates from the level flight assumption (Hedenström and Alerstam, 1992); and flock size, which introduces conflicting objectives between maximizing flight range and maintaining group cohesion (Hedenström and Åkesson, 2016, 2017a).

For isometrically scaled birds, any characteristic cruising flight speed is expected to increase as ∝*m*^1/6^ (where *m* is body mass) (Pennycuick, 1969), which appears to be valid in five near-isometric species of terns (Hedenström and Åkesson, 2016). However, when considering a larger sample of bird species, they depart from isometric scaling (Rayner, 1988) and, consequently, observed flight speeds increase with a slope lower than 1/6 (Alerstam et al., 2007; Pennycuick et al., 2013). In isometrically scaled birds, power required to fly increases as ∝*m*^7/6^, whereas the power available from the flight muscles, *P*_av_, increases less steeply at ∝*m*^2/3^ (Pennycuick, 1968), so that the margin between *P*_av_ and *P* declines with increasing body size (Pennycuick, 1975, 1987; but see Ellington, 1991). Consequently, the scope of achievable flight speeds may be restricted in very large birds.

Based on ecological objectives, we may expect different optimal speeds between seasons, for example, if autumn migration is energy selected (*V*_mr_) and spring migration is time selected (*V*_mt_>*V*_mr_) (Hedenström and Alerstam, 1995; Nilsson et al., 2013). Adaptive adjustments and external factors may require more or less power from the flight muscles than the derived characteristic speeds in still air. A relevant question in this context is whether constraints of muscle power may prevent large birds from achieving relevant predicted optimal speeds, and whether they are capable of adjusting speed to (head) winds adaptively. Previous studies have shown that many birds do adjust airspeed according to ecological context (e.g. Hedenström and Alerstam, 1996; Hedenström, 2024), and that realized flight speeds are influenced by a manifold of factors in terns and waders (Hedenström and Åkesson, 2016, 2017a; Hedenström, 2024).

The aim of this study was to test predictions about adaptive flight behaviour in a relatively large species, the greater cormorant *Phalcrocorax carbo sinensis* (henceforth ‘cormorants’ when referring to the birds of this study), where a limited scope owing to relatively small flight muscles may be expected, and yet high power requirements in flight owing to the relatively large size (body mass ∼2 kg). A study of the similarly sized Kergulean shag (*Leucocarbo verrucosus*) reported a relatively slow average airspeed at 12.7 m s^−1^, which was attributed to poor flight capacity owing to small flight muscles (Watanabe et al., 2011). European shags (*Gulosus aristotelis*) have likewise been observed to fly at relatively low airspeeds of 14.5 and 14.7 m s^−1^ (Pennycuick, 1987; Kogure et al., 2016), respectively. Pennycuick (1987) argued that observed airspeeds were only marginally higher than predicted *V*_mp_ and well below *V*_mr_, and attributed this to limited power available from the flight muscles. A reason for reduced flight muscle capacity in shags and cormorants could be that because of their foot propelled diving, flight muscles for aerial flight are traded off against leg muscles used when swimming and/or diving. Informed by flight mechanics and results from previous studies on shags and cormorants, we measured flight performance in relation to topography, ecological context, winds and flock size at a site in the southwest Baltic Sea with the aim of testing the following six predictions. (1) During autumn migration, we expect that cormorants RE not severely time-constrained and therefore prioritize transport economy, which implies their airspeed is *V*_mr_. Airspeed during spring migration and local commuting flights between nesting and foraging areas should be higher than *V*_mr_, assuming time-selected migration and maximization of the rate of energy transport to the nesting site, respectively (Norberg, 1981; Hedenström and Alerstam, 1995). Time-selected migration in spring is assumed based on the assumption of competition to reach favourable nesting sites before competitors, whereas during autumn migration the birds do not compete for such a specific resource. Test: *V*_spring_>*V*_autumn_ and *V*_summer_>*V*_autumn_. (2) If birds fly at *V*_mr_, they should adjust their airspeed according to head/tail winds, and to side winds if compensating for wind drift (Liechti et al., 1994). Airspeed should thereby be increased with increasing head-wind and absolute side-wind components and be reduced with increasing tail-wind component. Test: d*V*_a_/d*T*<0 or d*V*_a_/d*S*>0, where *T* and *S* are tail- and side-wind components, respectively. (3) If compensating for wind drift, birds should adjust their heading in parallel with speed adjustment. Strictly, partial drift/compensation occurs if *b*_track_>0, (see Eqn 2 below), but for the present analysis we consider that complete compensation for crosswinds is achieved if *b*_track_<0.1. (4) Airspeed should be adjusted in relation to vertical speed so that it decreases with increasing positive rate of climb and/or increases with increased rate of sink, provided the airspeed *V*>*V*_mp_ and *P*_av_>*P*(*V*) (Pennycuick, 1978; Hedenström and Alerstam, 1992). Test: d*V*_a_/d*V_z_<*0. (5) Under the assumption that characteristic flight speed increases with increasing body mass and that the flight speed of flocks is determined by the heaviest and fastest flying individuals of the flock (Hedenström and Åkesson, 2017a), there should be increased observed airspeeds with increasing flock size. Test: d*V*_a_*/*dlog*N>*0. And (6) The wind speed is lower at low altitudes owing to the friction-based wind gradient (Gauthreaux, 1991; Kemp et al., 2013); therefore, we expect that cormorants should fly at lower altitudes in head winds than otherwise. Test: d*z*/d*T*>0.

## MATERIALS AND METHODS

### Ornithodolite

We used an ‘ornithodolite’ (*sensu* Pennycuick, 1982), which in our case was identical to that used by Pennycuick et al. (2013). The ornithodolite setup consists of a Vector 21 Aero (Vectronix©) range finder, which is a 7×42 binoculars with built in sensors for azimuth and elevation angles and an infrared laser for measuring distance to a target (a bird or ascending balloon). The anemometer and sea surface heights in relation to the Vector were measured as part of the setup procedure at the start of an observation session. Individuals or flocks of birds were tracked by making a run of minimum two observations, each consisting of time-stamped distance, elevation and azimuth saved to a computer file. At the same time, wind strength and direction were measured using a Gill Windsonic ultrasound anemometer mounted on a 5-m extension mast placed in an unobstructed position near the ornithodolite. The wind data were transmitted to the computer at 1-s intervals via a pair of wireless modems (Haccom UM-96). For wind data at higher altitudes, we tracked ascending helium-filled balloons with the Vector at 1-h intervals, or more often if the wind direction or strength changed noticeably as indicated by the anemometer. The bird tracks represent the movement over the ground, and by using the ground speed vectors (*V*_g_) in combination with the wind vector (*V*_w_) measured at the relevant altitude of the flying bird, we obtained airspeed vectors (*V*_a_) by subtracting *V*_w_ from *V*_g_. The wind vector at altitudes above the anemometer threshold (15 m) was obtained by interpolating measurements of ascending helium-filled balloons to the relevant altitude where the bird was flying (Pennycuick et al., 2013). Wind measurements were corrected for surface friction in two steps according to the formula:
(1)

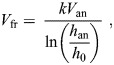
where *V*_fr_ is the friction wind speed, *V*_an_ is the anemometer reading at height *h*_an_ above the surface, *k* is von Karman's constant (0.42) and *h*_0_ is the roughness height (0.05 m). Wind speed *V*_w_ at the bird's measured height (*h*) was calculated as:
(2)

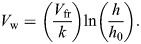


This gives a wind speed gradient, which starts at zero at height *h*_0_ above the surface, parses through *V*_an_ at the anemometer height and increases gradually above that height. Above the arbitrary chosen threshold (15 m), wind speed was obtained by interpolating measurements of ascending helium-filled balloons to the relevant altitude where the bird(s) was flying (Pennycuick et al., 2013). From the vectors *V*_g_ and *V*_a_, we obtained the associated track and heading. Birds were on average flying at distances of 549 m (range 37–2535 m) from the anemometer position. We calculated the mean speed and direction for each run, which constitute our observations that entered further analyses. In conjunction with the saving of a run, information about species (as we also measured species other than cormorants during field sessions), age composition and sex (when possible to determine), flight behaviour (continuous flapping, bounding, gliding, soaring, straight, meandering and circling) and flock size were noted. For more details about the ornithodolite setup, see Pennycuick et al. (2013).

### Field work

We tracked cormorants *Phalacrocorax carbo sinensis* (Staunton 1796) at two nearby sites on the island Öland in the southwestern Baltic Sea (Fig. S1): (A) east coast (56.250944°N, 16.484607°E) and (B) the southern point (56.209360°N, 16.405799°E). At site A, the coastline is oriented as 16 deg/196 deg, whereas at site B, the two coastlines converge towards the point of the island, with the west coast being oriented as 16 deg/196 deg and the east coast as 42 deg/222 deg. The distance between the two sites is 6.7 km and the direction from site B to site A is 226.5 deg. During migration at site B, the birds departed on an over-sea crossing towards southwest and thereby leave contact with coastlines until they reach the coast of the Swedish mainland on the other side of the sound between Öland and Sweden. Field work was carried out on 73 days distributed as March–May (site A: 2 days; site B: 20 days), June–July (site A: 8 days, site B: 2 days) and August–November (site A: 22 days; site B: 19 days). We referred to the tracked birds already in the field as being local foraging movements or migratory, based mainly on time of year and flock composition, as well as their behaviour. Flocks composed of only adult birds in early August were thereby classified as local movements, whereas if juveniles were in the flock they were considered as migrating. This is admittedly somewhat arbitrary, but we think the overall behavioural categories approximately reflect actual conditions.

Because this study involved remote sensing and no handling of live animals, no ethical permit was required according to Swedish legislation.

### Flight morphology

Body mass, wingspan and wing area were measured on seven individuals that had been captured in September 1992 by fishermen in their fishing tackle in Lake Vomb, southern Sweden (55.680403°N, 13.604180°E). Wingspan was measured between the wing tips on fully outstretched wings using a tape measure, and the wing outline of one fully outstretched wing was traced on paper. Wing area was then measured using a digital planimeter and total wing area estimated as twice the area of one wing plus the area of the body between the wing roots (Pennycuick, 2008). When the dead cormorants were processed at the Biological Museum, Lund University, the taxidermist dissected out and weighed the flight muscles of both sides of five of the birds (accession numbers L992/3431–35). The morphometry of seven adult cormorants, derived aspect ratio (wingspan squared divided with wing area) and wing loading (weight divided by wing area) are shown in Table S1.

### Predicted flight speeds

From the *P*(*V*) function it is possible to calculate the characteristic speeds *V*_mp_ and *V*_mr_ as follows (Pennycuick, 2008):
(3)

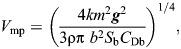

(4)

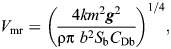
where *k* is the induced drag factor (=1.2 for flapping flight), *m* is body mass, ***g*** is acceleration due to gravity, ρ is air density, *b* is wingspan, *S*_b_ is body frontal area (=0.00813*m*^0.666^; Pennycuick, 2008) and *C*_Db_ is the body drag coefficient. Note that the formula for *V*_mr_ is for mechanical ‘ultimate maximum lift to drag ratio’, i.e. excluding profile power and any metabolic overheads. There is some controversy about which value should be assigned to *C*_Db_; therefore, we used two alternative values when calculating characteristic flight speeds. A value of *C*_Db_=0.2 is supported by wind tunnel measurements of mounted large waterfowl (Pennycuick et al., 1988) and recent measurements of drag in the wake of the free-flying jackdaw *Corvus monedula*, also in a wind tunnel (KleinHeerenbrink et al., 2016). A value of *C*_Db_=0.1 was suggested following observations of minimum wingbeat frequency of passerines and the teal *Anas crecca* flying in a wind tunnel (Pennycuick et al., 1996, 2012). In addition to calculations based on the formulae above, we also calculated *V*_mp_ and *V*_mr_ using a recent flight mechanical model (Fig. S2; https://github.com/MarcoKlH/afpt-r/; KleinHeerenbrink et al., 2015), which differs from the fixed wing model of Pennycuick (2008) by taking account of the aerodynamics of flapping wings. The calculated characteristic *V*_mp_ and *V*_mr_ are shown in Table 1.

**Table 1.** Predicted characteristic flight speeds based on two flight mechanical models

Model	*C*_Db_	*V*_mp_ (m s^−1^)	*V*_mr_ (m s^−1^)
Pennycuick (2008)	0.1	16.6	21.9
	0.2	14.0	18.4
	0.27	12.6	16.6
KleinHeerenbrink et al. (2015)	0.1	17.1	22.7
	0.2	15.3	20.1
	0.27	14.5	19.0

### Drift and statistics

Drift (*b*_track_) was calculated for autumn migrating cormorants at sites A and B using method 3 in Green and Alerstam (2002):
(5)

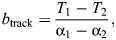
where *T*_1_ and *T*_2_ are track directions for the birds having the wind from the left and right with respect to the overall track direction for the whole sample, with *H*_1_ and *H*_2_ being the associated headings, and α_1_=*T*_1_–*H*_1_ and α_2_=*T*_2_–*H*_2_. If *b*_track_=0, there is full compensation and *b*_track_=1 implies full drift, whereas intermediate values represent partial drift/compensation. For the purpose of testing whether birds achieve compensation/drift, we consider evidence for complete compensation is sufficiently if *b*_track_<0.1.

Statistical analyses were performed using JMP Pro 17 (SAS Institute Inc.) and a linear model design with air speed (*V*) as the dependent variable, behaviour (spring migration, autumn migration and local movement) as a fixed category and other relevant variables as fixed effects nested within behaviour (see predictions above; Dataset 1). For circular data, we performed Watson–Williams *F*-tests for comparing means between circular distributions using Oriana^®^ version 4.

## RESULTS

### Flight directions and wind compensation

**Fig. 1.**
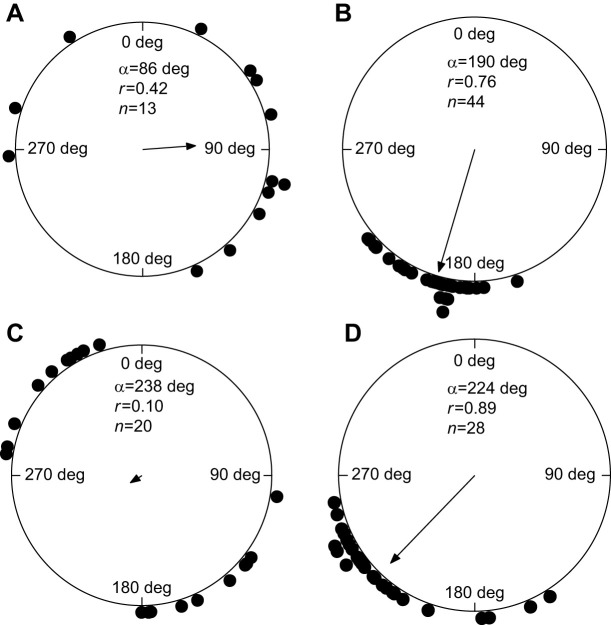
**Circular distributions of flight tracks in greater cormorants.** (A) Spring migration for sites A and B combined, (B) autumn migration at site A, (C) local summer flights at site B and (D) autumn migration at site B. α is mean direction, *r* is mean vector length, *n* is sample size.

Flights were highly oriented during autumn migration at both sites A and B, with a significant shift in mean track direction of 27 deg from 197 deg (site A) to 224 deg (site B) (Watson–Williams *F*_1,61_=24.5, *P*<0.001; Fig. 1B,D, Table 2). Spring migrants showed a more scattered orientation, with a mean direction towards east (Fig. 1, Table 2). Cormorants classified as being involved in local movements showed a similar orientation as autumn migrants at site A, but a bimodal orientation at site B (Fig. 1C, Table 2).

**Table 2.** Flight track directions at sites A and B during spring migration, autumn migration and summer local movements

	Site A			Site B		
Behaviour	Track (deg)	*r*	*n*	Track (deg)	*r*	*n*
Spring migration	190	1.0	2	75	0.33	11
Autumn migration	197	0.96***	35	224	0.89***	28
Local movements	190	0.76***	44	142^a^	0.68***	20

*r* is the vector length for circular data (range 0–1), *n* is sample size.

^a^Angular direction of axial distribution (142 deg/322 deg).

***P<0.001 according to the Rayleigh test (Batschelet, 1981).

At site A, the mean track direction of migrants and birds involved in local movements did not differ significantly (Watson–Williams *F*_1,77_=0.795, *P*>0.05; Table 2), and therefore we combined these data for the drift analysis. By subdividing the tracks into groups with winds coming from the left and right with respect to the overall mean track direction (194 deg), there was no significant difference between track directions (*T*_left_=195 deg, *N*=26; *T*_right_=194 deg, *N*=44; Watson–Williams *F*_1,68_=0.02, *P*>0.05), whereas the headings differed significantly (*H*_left_=188 deg, *N*=26; *H*_right_=203 deg, *N*=44; Watson–Williams *F*_1,68_=18.7, *P*<0.001; Fig. S3). This shows that birds compensate for wind drift, with a *b*_track_=0.035 indicating near-complete compensation. For site B, we could not combine data for local movement and migration because they were oriented differently, but for autumn migrants only the difference in heading between winds coming from left and right did not differ significantly (*H*_left_=220 deg, *N*=11; *H*_right_=231 deg, *N*=17; Watson–Williams *F*_1,26_=1.214, *P*>0.05), although the calculated amount of drift *b*_track_=0.40 indicates partial drift/compensation.

### Flight speeds

We analysed our data using a linear model including factors that may influence airspeed (dependent variable) with behaviour as a fixed category (spring migration, autumn migration, local movement), and the fixed factors (nested within behaviour) vertical speed (*V*_z_), tail wind component with respect to heading (*T*_comp,H_), side wind component (*S*_comp,H_), log_e_-transformed flock size and behaviour. Of all variables that were hypothesized to influence airspeed, only behaviour, vertical speed and tail-wind component had significant effects (Table 3). We removed non-significant factors until only significant factors remained in the model. Within behaviours, vertical speed was significant for spring migration only, whereas the tail-wind component was significant for local movements (with predicted negative slope of estimate) and spring migration (with positive slope of estimate) (Table S2B). A *post hoc* Tukey’s HSD test of pairwise differences indicated there was a significant difference in airspeed between the behavioural categories local movement and autumn migration (*t*=2.82, *P*=0.0153), and between spring and autumn migration (*t*=3.30, *P*=0.0036), but that there was no significant difference between spring migration and local movement (*t*=1.45, *P*=0.319). The measured airspeeds and associated confidence limits are shown in Table 4. We performed the same analysis, but with wind components projected onto the track directions instead of heading, with essentially identical results (Table S3).

**Table 3.** Outcome of linear model analysis of airspeed as the dependent variable, behaviour (spring migration, autumn migration, local movement) as the fixed category, and vertical speed (*V*_z_), tail-wind component with respect to heading (*T*_compH_), side-wind component (*S*_compH_), log_e_ of flock size as fixed factors (nested within behaviour) for greater cormorants

Source	d.f.	Sum of squares	*F*_2-3,132_	*P*
Behaviour	2	33.717	8.898	0.0045
*V*_z_	3	41.780	4.665	0.004
*T*_compH_	3	52.829	5.647	0.0008
*S*_compH_	3	19.479	2.175	0.0943
log_e_ Flock size	3	8.418	0.940	0.424

Effects of the source variables with wind components projected onto heading; *z* is altitude in meters, *V*_z_ is vertical speed (m s^−1^), behaviour is spring migration, autumn migration and local movement, *T*_compH_ is tail-wind component on heading, *S*_compH_ is side-wind component on heading.

**Table 4.** Measured airspeed (m s^−1^) for different behaviours (spring migration, autumn migration and local movement) in greater cormorants at Ottenby, southeast Sweden

Flight behaviour	*N*	Mean airspeed (m s^−1^)	s.d.	95% CI
Spring migration	13	18.85	2.82	17.76–19.94
Autumn migration	63	16.87	1.74	16.36–17.37
Local movement	64	17.95	1.81	17.44–18.46

### Flight altitude

**Fig. 2.**
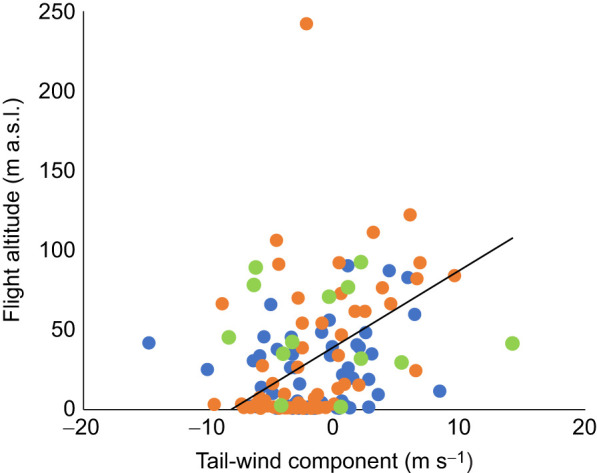
**Flight altitude (*z*) in relation to tail-wind component aligned with heading direction (*T*_comp,H_) for greater cormorants.** Summer data (blue) refer to local movements whereas spring (green) and autumn (orange) data refer to migration. The regression line (not shown in figure) has the equation: *z*(m)=4.80*T*_comp,H_+39.6.

We tested whether tail and side wind had an influence on the flight altitude according to prediction 6. There was an effect on flight altitude of tail-wind component (projected on heading and track, respectively), but it was significant only for autumn migration (Fig. 2; Table S4). During spring migration, the estimate had a negative slope (opposite to prediction; Table S4), which probably can be attributed to relatively low sample size.

## DISCUSSION

We start with an evaluation of the predictions put forward in the Introduction in relation to our data before discussing further the different aspects of flight speed adjustment and constraints in greater cormorants. Our hypothesis is that if flight muscle capacity limits the scope of flight performance, cormorants should not be able to adjust speeds as predicted. However, if a predicted adjustment is observed, flight muscle capacity is not limited.

### Evaluation of predictions

#### Prediction 1: *V*_spring_>*V*_autumn_; *V*_summer_>*V*_autumn_

Both spring migrating and summer commuting flights to foraging areas were faster than those of autumn migration. This suggests an influence of time-selected migration in spring compared with autumn, and an effect of foraging intake rate when flying between breeding and foraging sites (Hedenström and Alerstam, 1995).

#### Prediction 2: adjustment of airspeed to tail and side winds

Only the tail-wind component had a significant effect on airspeed, and only so during local movements. We therefore tentatively conclude that an adaptive speed adjustment according to tail winds is achieved.

#### Prediction 3: compensation for wind drift

Cormorants compensated for wind drift by adjusting heading direction when flying along a coastline (site A), otherwise they showed partial drift/compensation.

#### Prediction 4: effect of *V*_z_ on *V*

There was a significant effect of *V*_z_ on airspeed *V* only during spring migration, and so forward speed is traded against climb rate under some circumstances.

#### Prediction 5: *V* in relation to flock size

There was no significant effect of flock size on airspeed, and consequently the prediction was not supported.

#### Prediction 6: flight altitude in relation to wind direction

Flight altitude was affected by wind direction for local movements, with significantly lower flight altitudes in head-wind situations.

### Characteristic behaviour

Greater cormorants showed several adjustments of their flight behaviour in line with the predictions outlined in the Introduction (predictions 1–4, 6). Cormorants adjusted heading to compensate for wind drift when flying along a coastline, but showed partial drift when migrating away from landmarks at site B. This suggests that proximity to the coastline provides the birds with sufficient landmark reference to achieve complete drift compensation, whereas flying over a seascape far from land constrains the ability for complete wind compensation (cf. Alerstam and Pettersson, 1976). By flying at low altitude in headwind, the birds reduce the strength of the headwind they would otherwise meet at higher altitudes owing to the wind gradient in the surface layer (prediction 6; cf. Finn et al., 2012), which was the case during autumn migration. By flying low over a water surface, the birds can potentially also enjoy a reduced induced drag due to the ground effect (Blake, 1983; Rayner, 1991; Finn et al., 2012; Johansson et al., 2018). However, in tail wind, any benefit in ground effect from flying low is likely outweighed by the benefit of the wind support at higher altitude. The lack of a trade-off between airspeed and vertical speed during autumn migration and summer local foraging flights (prediction 4) was surprising considering the indisputable additional cost of climbing (Hedenström and Alerstam, 1992). We think this lack of expected dependency may be an effect of the narrow range of vertical speeds observed (only 26 tracks had |*V*_z_|>0.25 m s^−1^|), as most observations refer to birds flying at low altitude over water.

Adjustment of airspeed in relation to winds has been observed in the related European shag (Kogure et al., 2016). However, the predicted adjustment of airspeed with respect to sidewinds was not observed in cormorants, not even in situations where they clearly compensated for wind drift by adjusting the heading. We believe this is likely not related to sample size (type II error), as significant side wind effects have been detected in samples of similar or lower number of observations than that of the present study (see Hedenström et al., 2002; Hedenström and Åkesson, 2016, 2017a). It appears that the primary approach of cormorants to compensate for cross wind is by changing heading.

### Flight speeds: evaluation of *V*_mp_ and *V*_mr_

A first observation to make is that in previous studies on other cormorant species, the observed mean airspeeds were considerably lower than in our study (Table 6). Body mass (*m*) and morphological differences may explain this difference because any characteristic speed is expected to vary ∝*m*^1/6^ (ideal bird *sensu* Pennycuick, 1975). The scaling exponent for the species of Table 6 is 0.09 (*P*>0.05, *r*^2^=0.051). The mean airspeed of the Kerguelen shag is particularly low at 12.7 m s^−1^ (Table 6), which is possibly due to the method used to measure airspeed in that study (Watanabe et al., 2011). The bio-loggers were equipped with propellers, which, after calibration in a wind tunnel and operated in ‘air mode’, could be used to measure airspeed. It is possible that flow speed measured in this way could have been affected by the body and placement within the boundary layer. If excluding Kerguelen shag, the body mass scaling exponent becomes 0.29 (*P*<0.01, *r*^2^=0.98). Hence, the variation of airspeed among species is partly affected by body mass.

To find out why cormorants fly at the speeds they do, we will consider the characteristic speeds predicted by flight mechanical models and compare these with observed flight speeds. Comparisons between model-generated predictions and measurements of flight speeds in real birds ultimately depend on how well the model parameters can be estimated. One important parameter in these models is the body drag coefficient (*C*_Db_). A formula derived from wind tunnel measurements of large waterfowl and raptors yields a *C*_Db_ of 0.27 for greater cormorants at a Reynolds number of 162,000. A similar value of 0.28 was identified for Kerguelen shags (Watanabe et al., 2011). Measurements on a wingless peregrine body and a smooth model gave values for *C*_Db_ of 0.24 and 0.14, respectively (Tucker, 1990). Recent wind tunnel estimates of *C*_Db_ based on wake visualization for the jackdaw *C. monedula* suggested a value for *C*_Db_ near 0.2 (KleinHeerenbrink, et al., 2016), rather than 0.1 as suggested based on measurements of wing beat frequency in a few species (Pennycuick et al., 1996, 2012). While keeping in mind the substantial variation reported in the literature, we argue that inferences about flight speeds in large birds (>0.2 kg) are best based on the assumption that *C*_Db_=0.2. Therefore, *V*_mp_ should be ≤15.3 m s^−1^ (Table 1), whereas observed airspeeds in greater cormorants were significantly larger than 15.3 m s^−1^ (one-sample *t*-tests; autumn migration: *t*=7.22, *P*<0.0001; spring migration: *t*=4.42, *P*<0.0004; local movement: *t*=11.75, *P*<0.0001). Predicted *V*_mr_ for the model of KleinHeerenbrink et al. (2015) was 18.4 m s^−1^, whereas the observed airspeeds during autumn migration were significantly slower than this (one-sample *t*-test; *t*=6.89 *P*<0.0001) but observed speeds during spring migration were not (one-sample *t*-test; *t*=0.45, *P*>0.05). Airspeeds during local foraging flights were also significantly slower than 18.4 m s^−1^ (one-sample *t*-test; *t*=1.99, *P*=0.0256). This difference between spring and autumn could arise if birds are in worse condition during autumn migration because of a taxing breeding season. Alternatively, because cormorants typically use a flap–glide flight mode, another explanation could be that observed flight speed is a compromise between *V*_mr_ of flapping flight and the optimal speed of best glide (*V*_bg_=14.1 m s^−1^, assuming *C*_Db_=0.2 and morphology according to Table S1, calculated according to Pennycuick, 1989). On balance, the observed adjustment of airspeed to the tail-wind component supports the notion that cormorants usually fly at a speed ≠*V*_mp_, because *V*_mp_ should be unaffected by winds, and that observed speeds agree with the ecologically appropriate speeds (*V*_mr_, *V*_mt_ or *V*_opt_) associated with minimum cost of transport, time-selected migration or optimal speed between food patches (see Hedenström and Alerstam, 1995).

### Constraints on flight speed in cormorants?

To achieve a certain airspeed >*V*_mp_, the power delivered from the flight muscles must match the corresponding power required (Pennycuick, 1987). It has been argued that European and Kerguelen shags are unable to reach their respective *V*_mr_ due to insufficient muscle power (Pennycuick, 1987; Watanabe et al., 2011). The calculated maximum flight speeds with different assumptions of *C*_Db_, mass-specific muscle work and muscle mass for greater cormorants are shown in Table 5. To allow for sustained flight with the lowest muscle mass (10.3%) as found for the dead cormorants of this study, the models need *C*_Db_ to be as low as 0.1. But because we argued above that such a low value of *C*_Db_ is unlikely, it may be that the birds drowned in fishermen's tackle had unusually small flight muscle at the time of capture. For the flight muscle mass based on the only other empirical data for greater cormorants (13.6%), the flight at *V*_mr_ is feasible under most assumptions. If we focus on *C*_Db_=0.2, we see that this muscle mass brings the maximum achievable speed (22.2 m s^−1^) very close to the corresponding maximum range speed of 20.1 m s^−1^ (Table 1). Particularly, if we consider the muscle power density based on the model of Pennycuick and Rezende (1984), the maximum attainable speed (19.4 m s^−1^) is just below the predicted maximum range speed (20.1 m s^−1^). If we assume cormorants have a relative flight muscle mass of 17% (mean for all birds; Greenewalt, 1962), maximum speeds are generally beyond *V*_mr_, which would then suggest they were not constrained by available muscle power.

**Table 5.** Calculated maximum level airspeed and maximum range speed *V*_mr_ for a greater cormorant under different assumptions about muscle fraction, *C*_Db_ and muscle-mass-specific work

Muscle work	*C*_Db_	10.3%	13.6%	17%	*V*_mr_
100 W kg^−1^	0.1	–	20.1	25.5	22.7
	0.2	–	–	20.1	20.1
	0.27	–	–	17.1	19.0
140 W kg^−1^	0.1	22.0	27.4	31.3	22.7
	0.2	–	22.2	25.7	20.1
	0.27	–	19.8	23.3	19.0
Pennycuick and Rezende (1984)	0.1	17.5	24.8	28.4	22.7
	0.2	–	19.4	23.0	20.1
	0.27	–	16.3	20.6	19.0

Muscle work is based on Marden (1994), Pennycuick and Rezende (1984) and Hedenström and Alerstam (1992). Muscle fractions are based on the present study (10.3%) and Greenewalt (1962) (13.6%, 17%), where 13.6% refers to a greater cormorant and 17% is average for all birds. – indicates level flight is not possible at any speed.

**Table 6.** Measured mean flight speed in five species of cormorant based on published data, where *Phalacrocorax carbo* is represented by the present study

Species	Airspeed (m s^−1^)	Body mass (kg)	*N* (sample)	Source
*Nannopterum brasilianum*	14.2	1.3	15	Pennycuick (1983)
*Gulosus aristotelis*	15.4	1.81	103	Pennycuick (1987)
*Nannopterum auritum*	14.7	1.41	136	Pennycuick (1989)
*Leucocarbo verrucosus*	12.7	2.35	24	Watanabe et al. (2011)
*Phalacrocorax carbo*	17.4	2.55	140	Present study

Scientific names given here are those in current use for the relevant taxa, which differ from those in the original publications.

An additional diagnostic for a limited scope of flight speeds in cormorants is by comparing the coefficient of variation (100×standard deviation/mean, CV=11.3% in cormorants) with other species. The CV of airspeed was lowest for cormorants compared with nine other species (Table 7), indicating that the power scope is limited in relatively large birds such as cormorants. Finally, cormorants typically fly in flock formations, which may save energy for birds using a flap–gliding flight as cormorants (Weimerskirch et al., 2001). The combined effects of holding position in the formation and flock members being differently sized with different individual optimal flight speeds may lead to an overall compromise that masks adaptive speed responses as observed in some contexts. The resolution of flight speed selection in bird flocks clearly deserves further attention.

**Table 7.** Mean airspeed (*V*), standard deviation (s.d.), sample size (*N*) and derived coefficient of variation (CV) for 10 species of birds measured with the same Ornithodolite setup as used in the present study on greater cormorants

Species	*V* (m s^−1^)	s.d.	*N*	CV (%)	Source
*Apus apus*	12.0	2.49	141	20.7	Hedenström and Åkesson (2017b)
*Sterna paradisaea*	11.9	1.84	145	15.5	Hedenström and Åkesson (2016)
*Sterna hirundo*	11.7	1.94	82	16.6	Hedenström and Åkesson (2016)
*Sterna albifrons*	10.4	3.19	36	30.8	Hedenström and Åkesson (2016)
*Sterna sandwichensis*	12.6	2.25	56	17.9	Hedenström and Åkesson (2016)
*Calidris alpina*	16.1	2.40	161	14.9	Hedenström and Åkesson (2017a)
*Calidris canutus*	16.6	3.88	32	23.3	Hedenström and Åkesson (2017a)
*Haematopus ostralegus*	15.4	1.94	95	12.6	Hedenström and Åkesson (2017a)
*Phalacrocorax carbo*	17.5	1-98	140	11.3	Present study

## Supplementary Material

10.1242/jexbio.251056_sup1 Supplementary information

Dataset 1.
