# Supplementary material for: Flight performance of great cormorants Phalacrocorax carbo sinensis suggests sufficient muscle capacity for adaptive speed adjustment
Source: J Exp Biol. 2026 Jan 2;229(1):jeb251056. doi: 10.1242/jeb.251056 (PMC12813668; doi:10.1242/jeb.251056)
Supplement: Supplementary information [file jexbio-229-251056-s1.pdf]

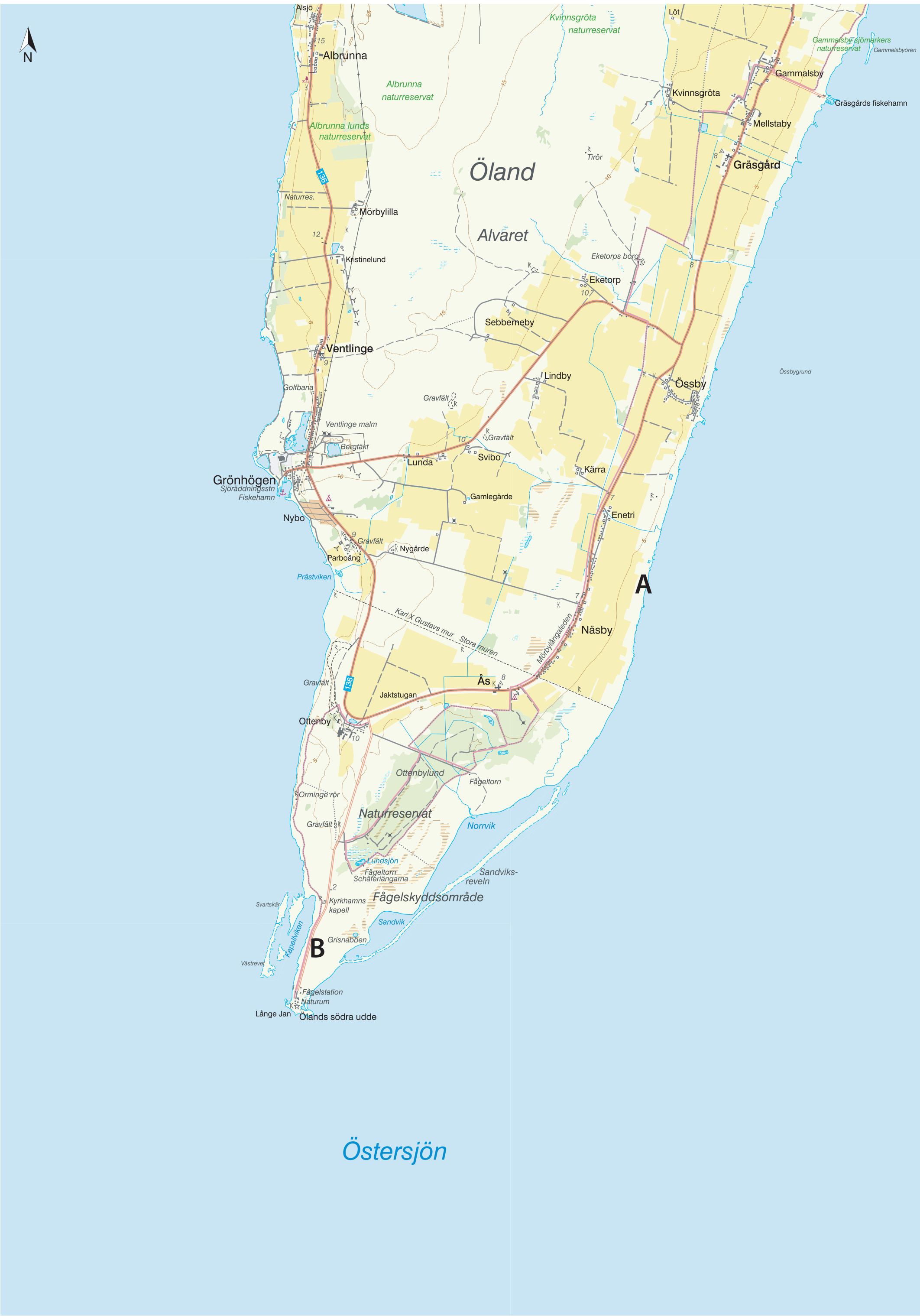

Skala 1:50 000, SWEREF 99 TM, RH 2000. Källa, Lantmäteriet http://www.lantmateriet.se/kartutskrift

Fig. S1. Map showing the southern part of the island Öland where observation sites A and B are indicated.

Journal of Experimental Biology • Supplementary information

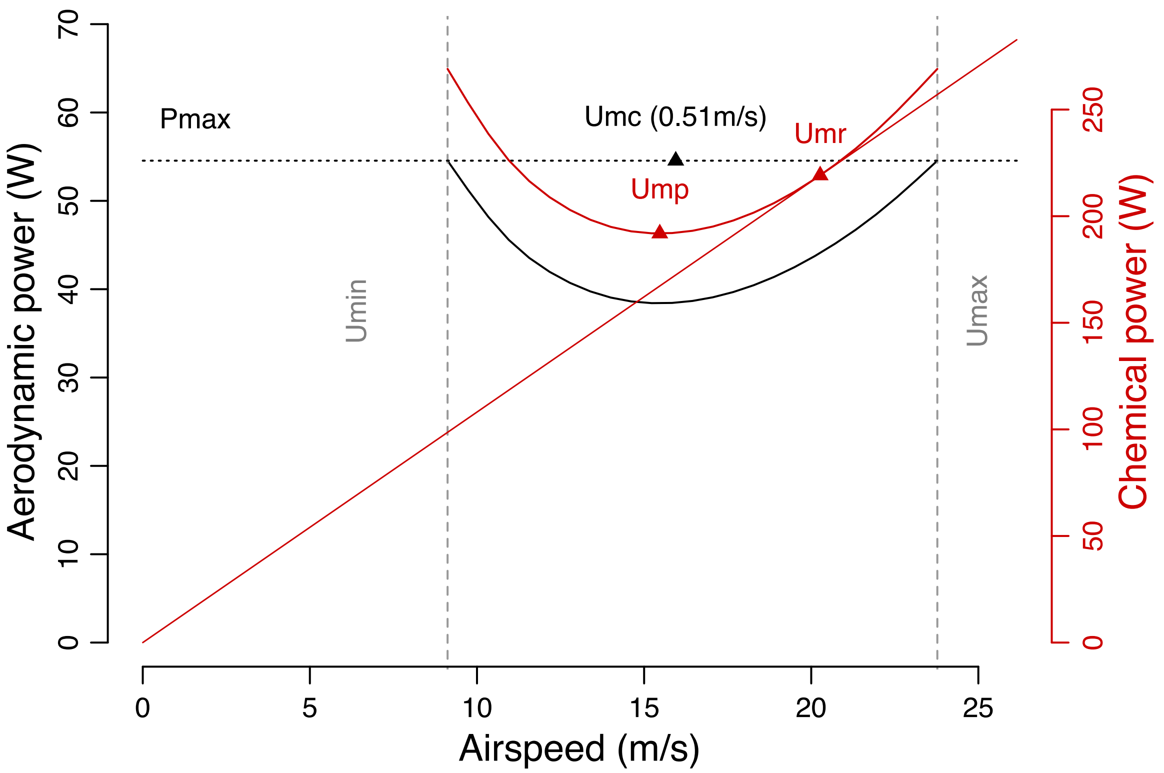

Fig. S2. Calculated power-airspeed relationships for a bird of the dimension of greater cormorant: body mass 2.5 kg, wing span 1.35 m, wing area 0.2242 m^2. Body drag coefficient was set to C_{Db}= 0.2. The lower (black) curve refers to mechanical power and the upper (red) curve is chemical power (assuming an energy conversion efficiency of 0.23). Calculated characteristic flight speeds are V_{mp} = 15.5 m/s, V_{mr}= 20.3 m/s, speed at maximum climb rate is 15.95 m/s and associated rate is 0.51 m/s. Minimum (9.1 m/s) and maximum (23.8 m/s) airspeeds are based on power available from flight muscles if muscle mass is 17% of total body mass calculated according to Pennycuick and Rezende (1984). Calculations were made using the R-software *afpt*, which is an implementation of the model by Klein Heerenbrink et al. (2015).

Journal of Experimental Biology • Supplementary information

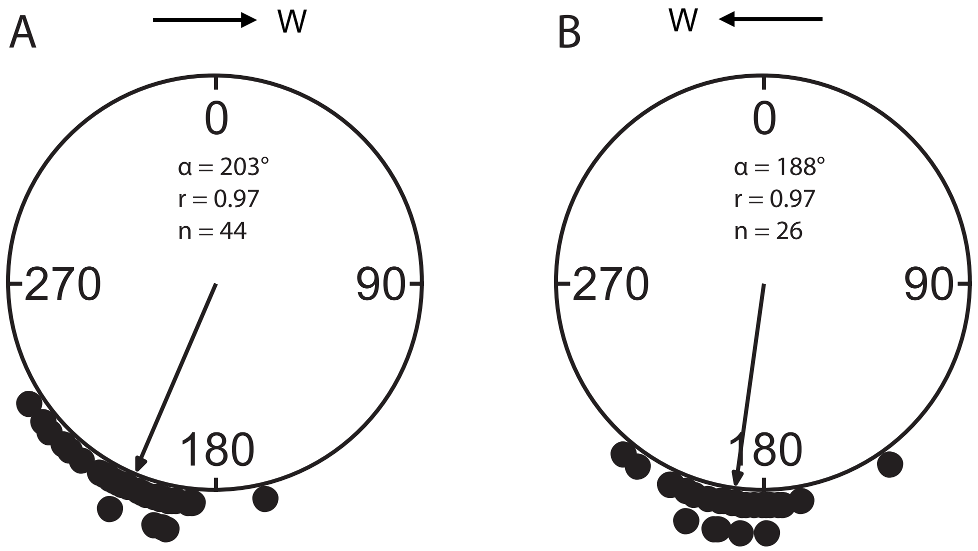

Fig. S3. Heading of greater cormorants at site A for summer local movements and migrating birds combined, where (A) shows heading for birds flying in winds coming from west, and (B) shows heading for birds flying in winds coming from east. The arrow above the diagrams indicate direction of wind (W). The difference between the two wind situations were significant (Watson-Williams test; $F_{1,68}$= 18.7, P<0.001), showing that the birds compensate for wind drift.

Table S1. Morphometrics of greater cormorants recovered by fishermen in lake Vombsjön (55.680403°N, 13.604180°E), Skåne, Sweden. Mass is total body mass, b is wing span, S is wing area including the projected area of the body between the wings, AR is aspect ratio, Q is wing loading, M_{musc} is mass of the flight muscles (pectoralis and supracoracoideus), and $M_{musc, rel}$ is proportion of flight muscle of the total body mass.

Bird#	Mass (kg)	b (m)	S (m^2)	AR (b^2/S)	Q (N/m^2)	M_{musc} (kg)	$M_{musc, rel}$ (%)
1	2.355	1.31	0.2036	8.43	113.5		
2	2.256	1.39	0.2291	8.43	96.6	0.2176	9.6
3	2.575	1.4	0.2521	7.77	100.2	0.2745	10.7
4	2.824	1.28	0.2316	7.07	119.6	0.2605	9.2
5	2.734	1.41	0.2314	8.59	115.9	0.3099	11.3
6	2.533	1.33	0.2047	8.64	121.4	0.2743	10.8
7	2.605	1.36	0.2169	8.53	117.8		
**Mean**	**2.555**	**1.35**	**0.2242**	**8.21**	**112.1**	**0.2674**	**10.3**

Journal of Experimental Biology • Supplementary information

Table S2. Result from a linear model with airspeed as dependent variable, behaviour (spring migration SM, autumn migration M, local movement LM) as fixed category, and vertical speed and tail wind component with respect to heading nested within behaviour. Minimal model with only significant factors included.

(A) Effect tests

**Source**	**Nparm**	**DF**	**Sum of squares**	**F**	**P**
Behavior	2	2	47.94932	7.8615	0.0006*
Uz[Behavior]	3	3	49.00417	5.3563	0.0016*
TcompH[Behavior]	3	3	57.98809	6.3382	0.0005*

(B) Parameter estimates

**Term**	**Estimate**	**SE**	**t**	**P**
Intercept	17.78508	0.194424	91.48	<.0001*
Behavior[LM]	0.172786	0.231745	0.75	0.4573
Behavior[M]	-0.881888	0.232426	-3.79	0.0002*
Behavior[LM]:(Uz+0.00842)	-1.21606	1.038934	-1.17	0.2439
Behavior[M]:(Uz+0.00842)	0.522107	1.05789	0.49	0.6225
Behavior[SM]:(Uz+0.00842)	-9.816432	2.581915	-3.80	0.0002*
Behavior[LM]:(TcompH+1.60743)	-0.176465	0.058974	-2.99	0.0033*
Behavior[M]:(TcompH+1.60743)	0.068779	0.058579	1.17	0.2425
Behavior[SM]:(TcompH+1.60743)	0.300004	0.101814	2.95	0.0038*

Table S3. Outcome of LM analysis of airspeed as dependent variable and behaviour (spring migration, autumn migration, local movement) as fixed category, vertical speed (V_z), tail wind component with respect to track (T_{compT}), side wind component (S_{compT}), $\log_e$ of flock size as fixed factors nested within behaviour for greater cormorants. N = 140.

(A) Effect tests for full model

**Source**	**DF**	**Sum of squares**	**F**	**P**
Behaviour	2	34.202	5.812	0.0039
V_z	3	35.964	4.074	0.0085
T_{compT}	3	45.579	5.164	0.0021
S_{compT}	3	19.332	2.190	0.0925
Log_e Flock size	3	11.218	1.271	0.287

(B) Effect tests for minimal model with only significant factors included

**Source**	**Nparm**	**DF**	**Sum of sqrs**	**F**	**P**
Behavior	2	2	52.90836	8.734	0.0003*
Uz[Behavior]	3	3	47.83427	5.2643	0.0018*
TcompH[Behavior]	3	3	60.71148	6.6815	0.0003*

(C) Parameter estimates for the minimal model

**Term**	**Estimate**	**SE**	**t**	**P**
Intercept	17.83123	0.191526	93.1	<.0001*
Behavior[LM]	0.10997	0.229126	0.48	0.6321
Behavior[M]	-0.939371	0.229644	-4.09	<.0001*
Behavior[LM]:(Uz+0.00842)	-0.739845	1.082012	-0.68	0.4953
Behavior[M]:(Uz+0.00842)	0.60975	1.065331	0.57	0.5681
Behavior[SM]:(Uz+0.00842)	-10.11608	2.61216	-3.87	0.0002*
Behavior[LM]:(TcompT+1.60743)	-0.214102	0.070582	-3.03	0.0029*
Behavior[M]:(TcompT+1.60743)	0.072614	0.055487	1.31	0.1929
Behavior[SM]:(TcompT+1.60743)	0.310965	0.102913	3.02	0.0030*

Journal of Experimental Biology • Supplementary information

Table S4. Outcome of LM of flight altitude (z) as dependent variable and tail wind component with respect to heading or track (T_{compH}, T_{compT}) as fixed factor nested within behaviour (spring migration SM, autumn migration M, local movement LM).

(A)

Effect tests

**Source**	**DF**	**Sum of squares**	$\mathbf{F_{1,134}}$	**P**
Behaviour	2	11044	5.124	0.0072
T_{compH}	3	23766	7.3512	0.0001

Parameter estimates

**Term**	**Estimate**	**Std error**	**t**	**P**
T_{compH} (LM)	1.295911	1.077839	1.2	0.2314
T_{compH} (M)	4.795073	1.060879	4.52	<.0001*
T_{compH} (SM)	-0.665999	1.57593	-0.42	0.6733

(B)

**Source**	**DF**	**Sum of squares**	**F**	**P**
Behaviour	2	10458	5.492	0.0051
T_{compT}	3	40589	14.210	<.0001

Term	Estimate	Std error	t	P
T_{compT} (LM)	2.614334	1.164253	2.25	0.0264*
T_{compT} (M)	5.730113	0.937954	6.11	<.0001*
T_{compT} (SM)	-0.764236	1.479943	-0.52	0.6064

Dataset 1. Cormorant data. The columns of the data file (.CVC) contain the following variables:

A Site, where A and B are sites defined in the Supplemental file
B Flock, is flock size
C E_{eq}, is equivalent airspeed (m/s)
D U, airspeed (m(s)
E H, Heading (degrees, north is 0)
F U_g, ground speed (m/s)
G T, Track direction (degrees, north is 0)
H U_w, wind speed (m/s)
I Wfrom, wind direction (degrees, north is 0), i.e. direction from which wind is blowing
J Wto, wind direction (degrees, north is 0), the direction to which the wind is blowing (Wfrom180)
K z, flight altitude
L U_z, vertical speed (m/s); positive for climbing, negative for descending
M Duration of track (s)
N Behaviour (LM is local movement, M is autumn migration, SM is spring migration)

Available for download at
https://journals.biologists.com/jeb/article-lookup/doi/10.1242/jeb.251056#supplementary-data
